# Introducing pulse oximetry in routine IMCI services in Bangladesh: A context-driven approach to influence policy and programme through stakeholder engagement

**DOI:** 10.7189/jogh.12.06001

**Published:** 2022-04-09

**Authors:** Ahmed Ehsanur Rahman, Sabrina Jabeen, Genevie Fernandes, Goutom Banik, Jahurul Islam, Shafiqul Ameen, Sabina Ashrafee, Aniqa Tasnim Hossain, Husam Md Shah Alam, Tamanna Majid, Ashfia Saberin, Anisuddin Ahmed, Ehtesham Kabir A N M, Mohammod Jobayer Chisti, Sabbir Ahmed, Mahbuba Khan, Tracy Jackson, David H Dockrell, Harish Nair, Shams El Arifeen, Muhammad Shariful Islam, Harry Campbell

**Affiliations:** 1 NIHR Global Health Research Unit on Respiratory Health (RESPIRE), Usher Institute, The University of Edinburgh, Edinburgh, UK; 2 icddr,b (International Centre for Diarrhoeal Disease Research, Bangladesh), Dhaka, Bangladesh; 3 Directorate General of Health Services, Ministry of Health and Family Welfare, Dhaka, Bangladesh; 4 Save The Children, Dhaka, Bangladesh; 5 World Health Organization Dhaka, Bangladesh

## Abstract

**Background:**

Pneumonia is the leading cause of under-five child deaths globally and in Bangladesh. Hypoxaemia or low (<90%) oxygen concentration in the arterial blood is one of the strongest predictors of child mortality from pneumonia and other acute respiratory infections. Since 2014, the World Health Organization recommends using pulse oximetry devices in Integrated Management of Childhood Illness (IMCI) services (outpatient child health services), but it was not routinely used in most health facilities in Bangladesh until 2018. This paper describes the stakeholder engagement process embedded in an implementation research study to influence national policy and programmes to introduce pulse oximetry in routine IMCI services in Bangladesh.

**Methods:**

Based on literature review and expert consultations, we developed a conceptual framework, which guided the planning and implementation of a 4-step stakeholder engagement process. Desk review, key informant interviews, consultative workshops and onsite demonstration were the key methods to involve and engage a wide range of stakeholders. In the first step, a comprehensive desk review and key informant interviews were conducted to identify stakeholder organisations and scored them based on their power and interest levels regarding IMCI implementation in Bangladesh. In the second step, two national level, two district level and five sub-district level sensitisation workshops were organised to orient all stakeholder organisations having high power or high interest regarding the importance of using pulse oximetry for pneumonia assessment and classification. In the third step, national and district level high power-high interest stakeholder organisations were involved in developing a joint action plan for introducing pulse oximetry in routine IMCI services. In the fourth step, led by a formal working group under the leadership of the Ministry of Health, we updated the national IMCI implementation package, including all guidelines, training manuals, services registers and referral forms in English and Bangla. Subsequently, we demonstrated its use in real-life settings involving various levels of (national, district and sub-district) stakeholders and worked alongside the government leaders towards carefully resuming activities despite the COVID-19 pandemic.

**Results:**

Our engagement process contributed to the national decision to introduce pulse oximetry in routine child health services and update the national IMCI implementation package demonstrating country ownership, government leadership and multi-partner involvement, which are steppingstones towards scalability and sustainability. However, our experience clearly delineates that stakeholder engagement is a context-driven, time-consuming, resource-intensive, iterative, mercurial process that demands meticulous planning, prioritisation, inclusiveness, and adaptability. It is also influenced by the expertise, experience and positionality of the facilitating organization.

**Conclusions:**

Our experience has demonstrated the value and potential of the approach that we adopted for stakeholder engagement. However, the approach needs to be conceptualised coupled with the allocation of adequate resources and time commitment to implement it effectively.

Pneumonia is the leading cause of childhood mortality, accounting for around 16% of 5.2 million under-five deaths globally [1-3]. In addition to mortality, childhood pneumonia contributes significantly to the global burden of disease through morbidities and long-term complications [4,5]. Moreover, most of these deaths and morbidities occur in low- and middle-income countries (LMICs) [1,2]. In Bangladesh, childhood pneumonia causes approximately 24 300 deaths per year, making it the leading cause of death in children younger than five years [6,7].

Hypoxaemia, defined by low (<90%) oxygen saturation in the arterial blood, is common among children with pneumonia and is one of the strongest predictors of mortality caused by pneumonia and other lower acute respiratory tract infections [8-12]. Early identification of hypoxaemia and timely administration of oxygen therapy can avert a significant portion of hypoxaemia-related deaths [8,10]. Pulse oximetry is an easy, non-invasive, and low-cost procedure used to measure and monitor the oxygen saturation level of arterial blood (SpO_2_) [13]. In high-income countries, pulse oximetry device is almost universally available as a standard for paediatric care but is rarely available and used in LMICs, particularly in routine outpatient services [11]. However, in 2014, the World Health Organization (WHO) revised its guidelines for routine outpatient services, ie, Integrated Management of Childhood Illness (IMCI), recommending the introduction and use of pulse oximetry in the early detection of hypoxaemia and referral decisions even in low-resource settings [12,14-16].

Bangladesh was one of the sites in the Multi-Country Evaluation of IMCI coordinated by WHO [17]. Based on the evidence generated from this study and global recommendations, the Government of Bangladesh adopted the IMCI strategy in 1998 as its primary approach to delivering outpatient-based services for sick children, including those with pneumonia. A decade later, by 2008, health facility-based IMCI was scaled up in all 64 districts and 420 of the 483 sub-districts (locally known as Upazilas) [18]. However, despite this progress, the use of pulse oximetry was still not recommended in routine IMCI services until 2018, as the assessment for pneumonia was primarily based on clinical history taking and physical examinations [19]. Therefore, an implementation research study was initiated in 2018 to assess the feasibility of introducing pulse oximetry in routine IMCI services in Bangladesh. The National Newborn Health and IMCI Programme of the Ministry of Health and Family Welfare led the study with technical support and facilitation from icddr,b (an international health research institute based in Bangladesh, which was formally known as International Centre for Diarrhoeal Disease Research, Bangladesh), and funding from the NIHR Global Health Research Unit on Respiratory Health (RESPIRE) through the University of Edinburgh, UK [20,21]. The implementation research was conducted in one district hospital, five sub-district hospitals (locally known as the Upazila Health Complexes) and five primary care health centres (locally known as Union Health and Family Welfare Centres) in Kushtia, a rural district in Bangladesh. The National Newborn Health and IMCI Programme in consultation with icddr,b selected Kushtia as the demonstration site as the under-five and neonatal mortality rates of the district were close to the average national estimates [22]. The objective of the study was to incorporate the recommendations for using pulse oximetry in routine IMCI services in national policy and programme documents, and assess the useability, acceptability, fidelity and utility based on WHO’s implementation research guideline [23].

Engaging a wide range of stakeholders is a key step for ensuring the relevance of research studies and improving the utilisation of research findings in local health systems settings [24,25]. As a result, health research funding agencies are increasingly promoting stakeholder engagement as an important step towards achieving impact [26]. Unfortunately, there is a dearth of evidence regarding the process of conceptualization, planning, implementation as well as mitigating challenges associated with stakeholder engagement in real-life settings. One of the main objectives of our implementation research was to promote context-specific solutions to implementation, participatory approach, government leadership, country ownership, scalability and sustainability by engaging key stakeholders at every stage of design, development, implementation and decision-making [25]. In this paper, we describe the rationale, strategy and process of influencing the policy and programme through a context-driven stakeholder engagement approach.

## METHODS

### Approach-adaptation of a conceptual framework for stakeholder engagement

**Figure 1:**
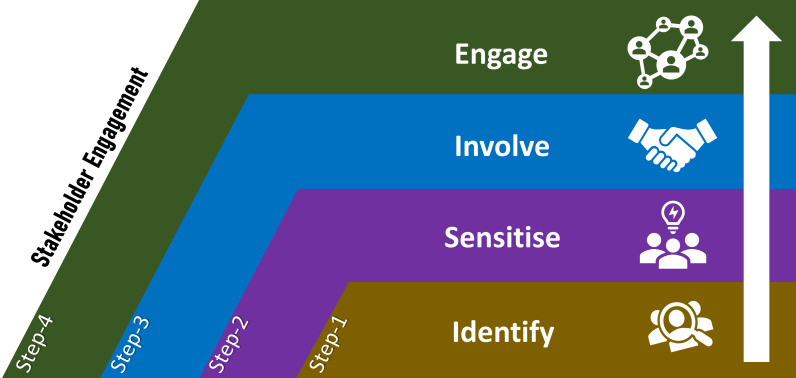
Conceptual framework adapted to guide the stakeholder engagement process to integrating pulse oximetry in routine Integrated Management of Childhood Illness (IMCI) services in Bangladesh.

Stakeholders are individuals, organisations, or communities that can influence or are affected by a project's process and outcomes, research or policy [27]. Stakeholder engagement is an iterative process of actively soliciting interested and influential individuals' knowledge, experience, and values to create a shared understanding, mutually agree on outcomes, and make relevant, transparent, and effective decisions. Essentially, a bi-directional relationship between the stakeholders and the researchers results in informed decision-making about the prioritisation, implementation, and use of research findings [28]. The literature on stakeholder engagement in health research points towards various processes and approaches, primarily derived from priorities, opportunities and contexts [24,28-30]. Guided by the literature, consultations with policymakers and experts, and based on practical experiences in implementing development projects in the context of Bangladesh, we adapted a conceptual framework to guide the stakeholder engagement related activities in our study (**Figure 1**). This framework proposes four steps. **Step-1:** Identification and prioritisation of stakeholders for subsequent sensitisation, involvement and engagement. **Step-2:** Sensitising the majority of stakeholders regarding the context, rationale, importance and process of the proposed research to build interest, confidence and trust. **Step-3:** Involving key stakeholders in planning, ensuring agreement regarding the implementation process and solidifying commitments for shared responsibilities. **Step-4:** Engaging the stakeholders at every stage of design, development, implementation and evaluation.

### Process steps taken to implement stakeholder engagement related activities

#### Step 1: Identifying stakeholders for sensitisation, involvement, and engagement

In Bangladesh, health services are provided by two directorates under the Ministry of Health and Family Welfare. They are the Directorate General of Health Services and Directorate General of Family Planning. Each directorate has a dedicated programme for child health, which manages the child health services, including IMCI services in Bangladesh. In addition, several other programmes are also related to child health services. Besides the government programmes, several development partners and professional societies support the Government of Bangladesh in managing and implementing child health services, including IMCI services. The National Newborn Health and IMCI Programme under the Directorate General of Health Services of the Ministry of Health and Family Welfare is responsible for coordinating with all partners and ensuring the delivery of IMCI services in Bangladesh.

First, comprehensive desk review was conducted on IMCI related documents and eight key informant interviews to identify national and district level stakeholders related to IMCI implementation in Bangladesh (Table S1 and S2 in the **Online Supplementary Document**). We purposively selected the key informants based on their former and current involvement with the IMCI programme in Bangladesh. After reaching reasonable level of data saturation, we identified 15 national-level and 16 district-level stakeholder organisations ranging from government health programmes, professional societies, UN agencies, local, national, and international non-government organisations (NGO), health service providers, etc. As Kushtia was our demonstration district, we identified the IMCI related stakeholder organisations exclusively working in Kushtia district.

We primarily focused on influencing the policy and guidelines to introduce and integrate pulse oximetry in routine IMCI services in Bangladesh. We also wanted to maximise the use of limited resources by prioritising a few stakeholder organisations that we would engage with. Hence, we organised a consultative workshop with the Programme Manager and all of the five Deputy Programme Managers of the National Newborn Health and IMCI Programme, where we used the Power-Interest matrix for stakeholder prioritisation [31].

**Figure 2:**
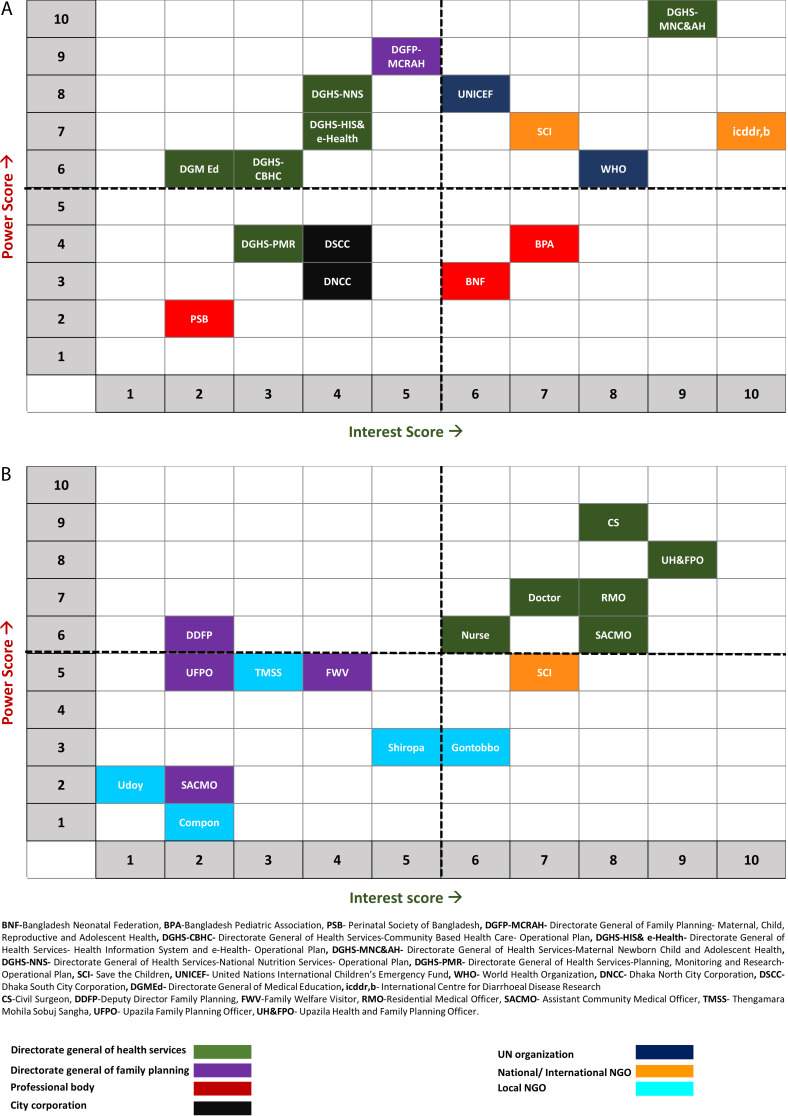
Power-Interest mapping of national and district level stakeholder organisations related to Integrated Management of Childhood Illness (IMCI) implementation in Bangladesh.

At the workshop, the participants scored each stakeholder organisation on their level of power and interest regarding IMCI implementation in Bangladesh based on a 10-point Likert scale where one was regarded as the lowest and ten was considered the highest (Table S3 in the **Online Supplementary Document**) [32]. We then plotted each stakeholder organisation (identified through desk review and key informant interview) in the Power-Interest matrix based on their average scores allotted by the workshop participants (**Figure 2**). Any stakeholder organisation with an average interest score of 6 and above was considered to have high interest. Similarly, any stakeholder organisation having an average power score of 6 and above was considered to have high power. Thus, of the 15 stakeholder organisations identified at the national level, five were categorised in the high power-high interest group, five in the high power-low interest and two low power-high interest groups, while four stakeholders were classed into the low power-low interest group. The 16 district-level stakeholder organisations were categorised similarly, with six prioritised as having high power-high interest. We decided to inform (described in step 2) all the stakeholders with high power or high interest but focus our attention on stakeholders with high power-high interest during the involve (described step 3) and engage (described in step 4) phases.

#### Step 2: Sensitising stakeholders for awareness building and understanding

Two sensitisation workshops were conducted with national-level stakeholders prioritised in the previous step. In the first workshop, we invited 40 participants from stakeholder organisations representing the high power-high interest, high power-low interest, and high interest-low power groups. The Programme Manager of the National Newborn Health and IMCI Programme chaired the workshops to promote country ownership and government leadership. In the first workshop, the research team of icddr,b presented the burden of hypoxaemia among children suffering from pneumonia, the importance of using pulse oximetry in hypoxaemia assessment and the updated WHO recommendation for introducing pulse oximetry in routine assessment and classification of pneumonia. In the second workshop, the invitation was limited to 19 participants from five organisations representing the high power-high interest group [14,15,33,34]. The participants discussed both the technical and operational feasibility of introducing pulse oximetry in routine IMCI services in Bangladesh.

A sensitisation workshop was conducted in Kushtia, with 32 participants from all district level stakeholder organisations, including the district health manager (locally known as Civil Surgeon, ie, CS), district level family planning manager (locally known as Deputy Director Family Planning, ie, DDFP), all (five) sub-district health managers (locally known as Upazila Health and Family Planning Officers, ie, UH&FPO) and all sub-district level family planning managers (Upazila Family Planning Officers, ie, UFPO). In addition, managers from the National Newborn Health and IMCI Programme attended the event. They presented the importance of identifying hypoxaemia in pneumonia assessment and classification and discussed the operational feasibility of introducing pulse oximetry in real-life settings.

Subsequently, we organised five such sensitisation workshops in each sub-district, with 14 participants in each workshop, including IMCI service providers such as doctors, nurses and paramedics (locally known as Sub-assistant Community Medical Officers, ie, SACMO) and their respective supervisors. For each sub-district level workshop, the respective sub-district health managers gave the keynote presentation.

Lastly, 71 enlisted national and local level NGOs actively working in Kushtia district were identified, of which 21 had strong community acceptance and health-related activities. We organised another sensitisation workshop with representatives from these 21 organisations. The district health manager gave the keynote presentation, and all the sub-district managers contributed to the subsequent discussions. All participating NGO partners agreed to raise awareness regarding pneumonia and hypoxaemia and promote appropriate care-seeking practices in the local communities.

#### Step 3: Involving stakeholders in joint planning and sharing responsibilities

Like the previous sensitisation phase, we organised workshops at the national, district, and sub-district levels to involve stakeholder organisations representing the high power and high-interest group, discuss a strategy, and develop a mutually agreed plan for introducing pulse oximetry in routine IMCI services of Bangladesh. At the national level, the Programme Manager of the National Newborn Health and IMCI Programme chaired the workshop. All the participants discussed and developed a national action plan to introduce pulse oximetry in routine IMCI services in Bangladesh. The roles of all key stakeholder organisations were clarified and defined in this comprehensive action plan. The National Newborn Health and IMCI Programme agreed to lead this process, and WHO, UNICEF, Save the Children and icddr,b committed to providing technical assistance and implementation support. The National Newborn Health and IMCI Programme also decided to update the National IMCI Implementation Package based on the updated WHO recommendations, including introducing pulse oximetry in pneumonia assessment and classification. This National IMCI Implementation Package consists of guidelines, registers, referral forms, reporting forms, job aids, monitoring checklist, list of essential equipment, medicine and logistics and training manuals. It was also decided that the development partners will be initially responsible for the procurement and supply of pulse oximetry devices in some demonstration facilities. The National Newborn Health and IMCI Programme declared to include pulse oximeters in the national procurement plan if the demonstration is successful.

A small working group was formed with technical experts from each stakeholder organisation with high power-high interest to update the implementation package. The small working group recommended assessing the feasibility of implementing the updated implementation package (including pulse oximetry) through an implementation research study. icddr,b volunteered to lead this implementation research study in Kushtia district. The working group members identified the evidence gaps and finalised key research questions for the implementation research through a consultative process. Further, at this workshop, it was also decided that the experience gathered through this implementation research would assist the Government of Bangladesh in taking evidence-based decisions for a national scale-up.

A planning workshop was organised in Kushtia district headquarter, which was chaired by the district health manager and attended by all sub-district health managers. At this workshop, the participating stakeholders decided that the implementation research would include all types of health facilities providing IMCI services. Hence, the Kushtia District Hospital, all five Upazila Health Complexes and one Union Health and Family Welfare Centre from each sub-district were selected as demonstration sites for the implementation research study. A district-level action plan was jointly developed outlining the roles of different district level stakeholders and the key activities, including training, distribution of pulse oximetry device, revised IMCI registers, referral forms and reporting forms, supportive supervision visits and assessments. Subsequently, a planning workshop was organised in each sub-district with the supervisors of IMCI service providers. The respective sub-district managers chaired this event and shared guidance on developing a sub-district level implementation plan for introducing pulse oximetry. A preparatory and planning meeting followed in each demonstration facility with IMCI service providers, respective supervisors, and facility managers, leading to the finalisation of a facility-specific implementation plan (with a timeline) to introduce pulse oximetry minimally impacting their routine services and existing practices.

#### Step 4: Engaging stakeholders in design, development, and implementation

In this stage, National Newborn Health and IMCI Programme and icddr,b worked closely with the high power-high interest stakeholders on the following steps to update the IMCI implementation package and implement it in the demonstration facilities in real-life settings.

**Figure 3:**
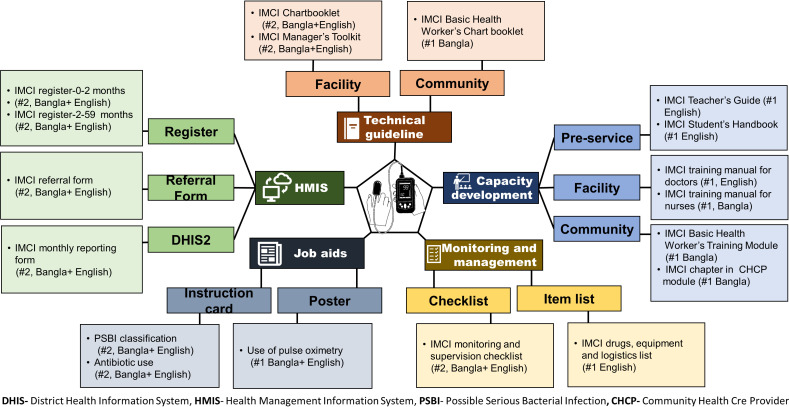
Documents in the National Integrated Management of Childhood Illness (IMCI) Implementation Package updated to incorporate the recommendation for using pulse oximetry in routine IMCI services.
- **Orientation training of national IMCI master trainers and pre-testing with IMCI service providers:** We organised a workshop to orient the national IMCI master trainers to the updated National IMCI Implementation Package, including the use of pulse oximetry in pneumonia assessment and classification. The master trainers reviewed the updated documents (primarily the chart booklet and training manuals) and shared their feedback.

- ***Formation of a working group:*** The Line Director of Maternal, Newborn, Child and Adolescent Health Programme of the Ministry of Health and Family Welfare formed a small working group with 13 IMCI experts representing the five high power-high interest groups to review and update the National IMCI Implementation Package (Table S4 in the **Online Supplementary Document**). The Programme Manager of the National Newborn Health and IMCI Programme chaired this group and icddr,b provided secretarial support. The group agreed to review the current availability of pulse oximetry devices in public health facilities and the overall oxygen delivery system in the country in addition to incorporating the recommendations and instruction to use pulse oximetry in the national IMCI implementation package.
- **Updating the National IMCI Implementation Package:** The working group reviewed the National IMCI Implementation Package, which was based on the previous WHO guideline in 2009 and compared them with the updated WHO guidelines in 2014 [15,35] (Table S5 in the **Online Supplementary Document**). In addition to the new guidance on using pulse oximetry in pneumonia assessment and classification, WHO recommended other major changes in the assessment, classification and management of possible serious bacterial infection (among children aged 0-59 days), pneumonia (among children aged 2-59 months), diarrhoea, dehydration and malnutrition. The working group discussed these changes and decided to incorporate them in the national IMCI guidelines based on the country context, rationale, and operational feasibility. Based on these decisions, the working group also updated the national IMCI chart booklet of Bangladesh. The key changes that are incorporated in the National IMCI chart booklet and summarised in Table S6 in the **Online Supplementary Document**. Subsequently, relevant training manuals were updated for IMCI services providers (doctors, nurses, and paramedics), community health workers and medical students (pre-service training). The working group also updated the IMCI-related service register, referral form, reporting form and job aids. Additional implementation materials such as the essential list of IMCI-related equipment, drugs and logistics, and monitoring checklists were also updated. All the National IMCI Implementation Package documents were developed in English and Bangla (the official language of Bangladesh). It took the working group a total of thirteen meetings (each lasting from three to five hours) to review and update all the documents in the IMCI implementation package (**Figure 3**).

**Figure 4:**
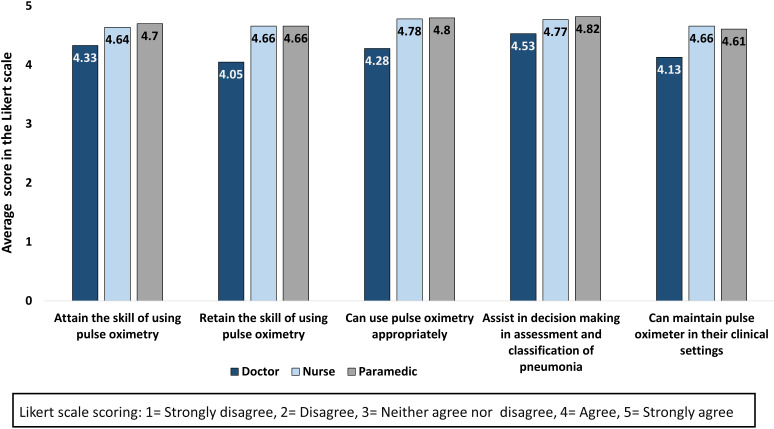
Response of Integrated Management of Childhood Illness (IMCI) service providers regarding perceived importance, acceptance and confidence of using pulse oximetry after receiving training with the updated implementation package, presented in average score in a five-point Likert scale, doctors = 40, nurses = 91, paramedics = 67.

Afterwards, the updated training package was pre-tested among IMCI service providers, including doctors, nurses, and paramedics. The National Newborn Health and IMCI Programme organised the training, the national master trainers facilitated the sessions, and icddr,b conducted assessment and evaluations. A total of 40 doctors, 91 nurses and 67 paramedics received training in 10 batches. We collected feedback from the IMCI providers on the National IMCI Implementation Package, including the training manuals, registers, job aids and reporting forms. **Figure 4** shows the response of the IMCI service providers regarding the perceived importance, acceptance and confidence of using pulse oximetry in a five-point Likert scale, which were collected after the end of the training. The average Likert scale score was more than four across all response categories. We also did not find any significant difference (ANOVA test) across the three types of providers for any of the response categories.

- **Finalisation of the National IMCI Implementation Package:** Following the training workshops with master trainers and IMCI service providers, the working group updated the national implementation package based on their feedback. Then National Newborn Technical Working Committee for Newborn Health and the National IMCI Technical Working Committee, the highest-level technical committees regarding newborn and child health in Bangladesh, reviewed the updated package and shared feedback. The National IMCI Implementation Package was finalised after incorporating their feedback and inputs.
- **Incorporation of the use of pulse oximetry and hypoxaemia in the national health service records:** Over a series of six consultative workshops, the National Newborn Health and IMCI Programme convinced the Management Information System (MIS) department of the Directorate General of Health Services to update the national IMCI dataset (maintained in the national DHIS2) based on the updated monthly reporting form (including updated pneumonia classification and hypoxaemia status). icddr,b provided technical assistant and secretarial support in organising these events.
- **Involving national-level stakeholders in the implementation research:** A co-design and co-creation approach was adopted to develop the implementation research protocol. icddr,b led the process and engaged the National Newborn Health and IMCI Programme and other stakeholder organisations representing the high-power and high-interest group to prioritise research questions and finalise the research design, data collection methods, and data collection instruments. Additionally, one of the Deputy Programme Managers of the National Newborn Health and IMCI Programme joined the research team as a Co-Investigator.
- **Local-level involvement in the implementation research:** A district advisory committee was formed to guide the implementation research study in Kushtia-district. The district health manager chaired the committee, and all the sub-district level health managers were included as members. First, the advisory committee was oriented regarding the research questions and study design. Later, the committee developed a macro plan to introduce pulse oximetry in the selected facilities and conduct quantitative and qualitative assessments as outlined in the research protocol. Subsequently, a planning meeting was organised in each facility with respective IMCI service providers and their supervisors to review the macro plan and develop a micro plan with a timeline. A dissemination meeting was organised with the district advisory committee after the baseline and each round of data collection. icddr,b provided technical assistance and secretarial support throughout the process.
- **Resuming study activities during the COVID-19 pandemic:** The Government of Bangladesh declared a nationwide lockdown in the last week of March 2020 to prevent the surge of COVID-19 infections and deaths. Unfortunately, all the implementation research-related activities were suspended due to the strict restrictions imposed during the lockdown period. In August 2020, the government started to reduce restrictions. The National Newborn Health and IMCI Programme issued an official order to resume the research activities with appropriate infection prevention and control measures. A series of meetings were organised in Kushtia-district with the district health manager and sub-district health managers to resume the suspended activities and revise the implementation plan (both macro plan and micro plan) accordingly.

### Resources required for stakeholder engagement

**Figure 5:**
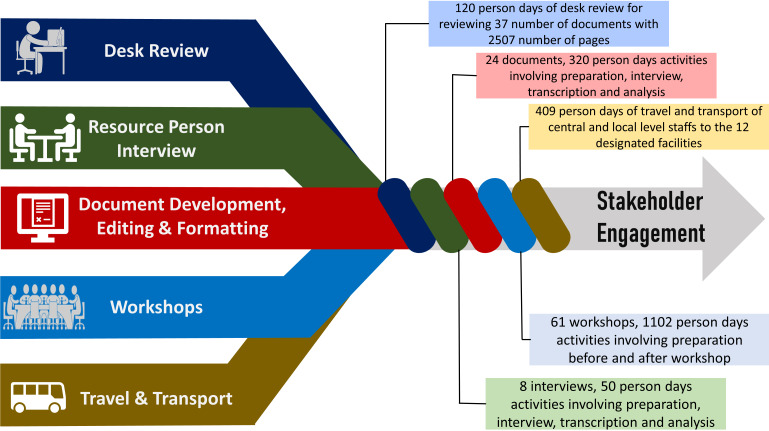
Resources required and invested for stakeholder engagement activities.

**Figure 5** summarises the resource required for undertaking the above-mentioned stakeholder engagement related activities.

**Desk Review:** A total of 37 documents with approximately 2507 pages were reviewed to identify the stakeholders and update the National IMCI Implementation Package. It required around 120 person-days of effort by medical graduates with basic IMCI training.

**Resource person interview:** A total of eight interviews and a group discussion was organised with IMCI experts to identify and prioritise the stakeholder organisations, requiring ten person-hours for interviewing and an additional 30 person-hours for transcribing and analysis.

**Document development, editing and formatting:** The National IMCI Implementation Package containing 24 documents with 1685 pages were updated, which required approximately 140 person-days of reviewing, editing and formatting to prepare the final versions.

**Workshops:** At the national level, 30 workshops were organised to sensitise (step 2), involve (step 3) and engage (step 4) the stakeholders, requiring approximately 416 person-days of involvement by IMCI experts and stakeholders. In addition, about 42 person-days of investment was needed from icddr,b for taking preparation and organising the events. Furthermore, around 30 person-days was required to prepare and approve the meeting notes for official documentation.

At the district level, 45 meetings were organised, requiring approximately 183 person-days of involvement by IMCI service providers, 86 person-days by their clinical supervisors, and 101 person-days by district, sub-district and facility managers.

**Travel and transport:** To sensitise (step 2), involve (step 3) and engage (step 4) stakeholders at the district level, extensive travel and transport were required. A total of 97 person-days of travel and transportation were needed from central-level staff and 312 person-days from local-level staff to organise and participate in the stakeholder engagement related events

### Timeline

**Figure 6:**
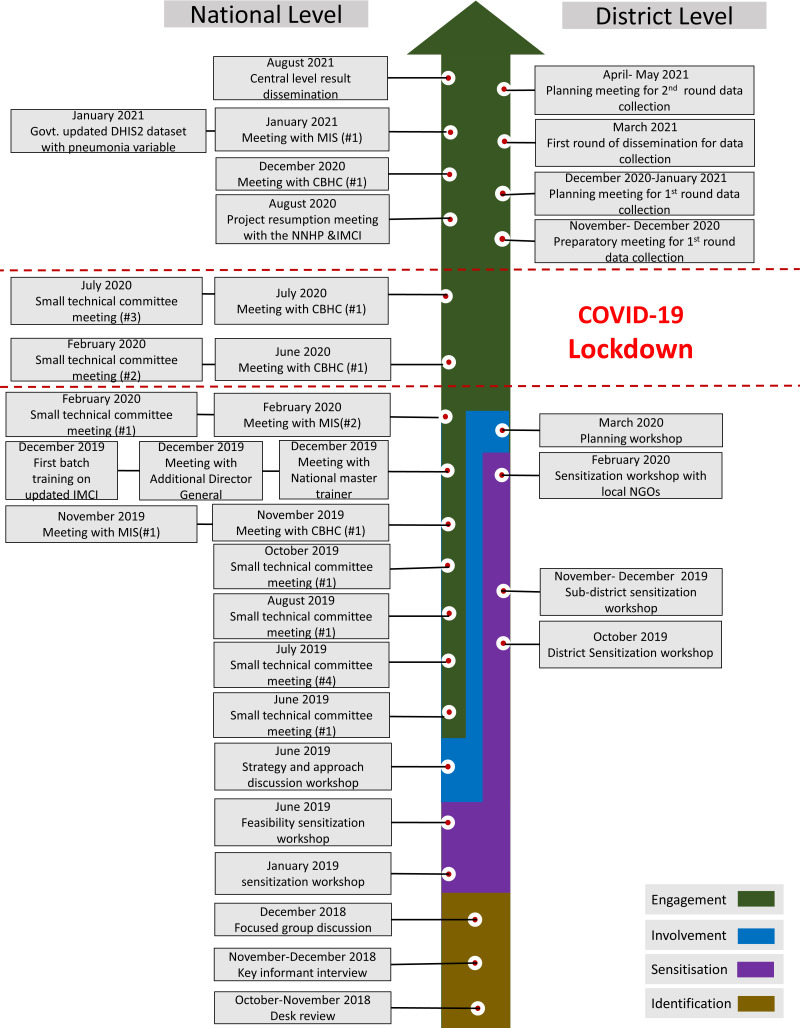
Milestones and timeline of stakeholder engagement activities.

**Figure 6** presents the timeline of major stakeholder engagement related activities.

**Step 1-Identify:** This process began in October 2018 and continued till December 2018.

**Step 2- Sensitise:** At the national level, the sensitisation process started in January 2019 and continued till the end of that year, while at the district level, the sensitisation activities went on from October 2019 till February 2020.

**Step-3-Involve:** At the national level, the involvement process took place in June 2019 and at the district level, the process took place in March 2020.

**Step 4-Engage:** The national-level engagement process started in June 2019 and ended in January 2021. The district-level engagement process began in December 2020 and ended in July 2021.

## RESULTS

### Impact of the stakeholder engagement process

#### Change in national policy and strategy

The Government of Bangladesh and all the key stakeholders acknowledged the importance of identifying hypoxaemia through SpO_2_ assessment for improving pneumonia assessment, classification, and management. As a result, the Government of Bangladesh decided to introduce pulse oximetry in routine IMCI services in Bangladesh.

#### Change in the national programme

The National Newborn Health and IMCI Programme formed a national-level working committee with high power-high interest stakeholder organisations related to IMCI implementation in Bangladesh. This high-level working committee updated the National IMCI Implementation package including the chart booklet, register, referral form, reporting form, monitoring checklist and training manuals and incorporated specific recommendations to use pulse oximetry in relevant sections. Further, three hypoxaemia related indicators were included in the national IMCI data set in DHIS2. The Government of Bangladesh trained 4872 IMCI service providers from across the country based on the updated National IMCI Implementation Package.

#### Progress towards scalability and sustainability

The Government of Bangladesh decided to scale up the use of pulse oximetry in routine IMCI services in a phased manner. As a result, the National Newborn Health and IMCI Programme revised its existing plan and decided to procure pulse oximetry devices for the IMCI corners in approximately 200 Upazila health complexes. In the next phase, the National Newborn Health and IMCI Programme is planning to procure pulse oximetry devices for the remaining of the IMCI corners in the Upazila Health Complexes (approximately 250) and other union-level facilities (approximately 5000). Additionally, UNICEF has committed to support the Government of Bangladesh’s national scale-up endeavour by introducing pulse oximetry in eight districts. icddr,b has committed to support in two districts, and Save the Children in one district. Moreover, WHO has supported the government by printing the updated IMCI registers for all facilities.

### Lessons learned

**Figure 7:**
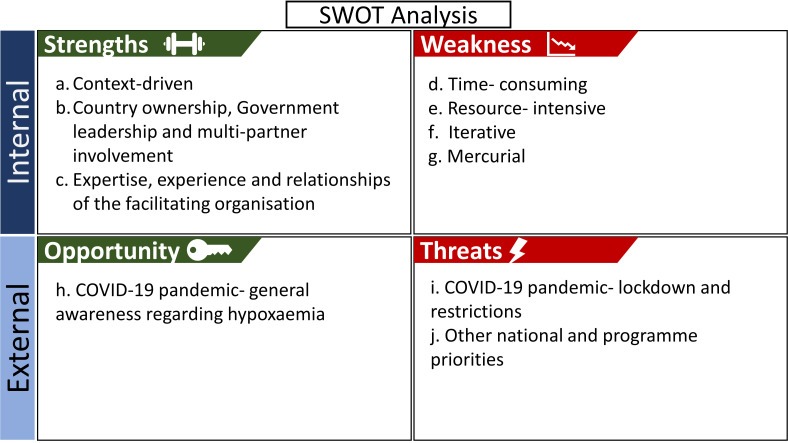
Strength, weakness, opportunity, and threat analysis of the stakeholder engagement process.

We present the key learnings from the stakeholder engagement activities based on the SWOT analysis approach [36,37]. The summary of the SWOT analysis is presented in **Figure 7****.**

#### Strength

- **Context-driven:** Our implementation research study primarily aimed to influence the national policy and programmes. Based on the context and prior experience, we adopted a framework with four steps for stakeholder engagement and prioritised only the high power-high interest stakeholder organisations. The framework guided the whole process and all related activities, and the prioritisation helped maximise the use of limited resources. Therefore, any implementation research study aiming to engage stakeholders need to adapt and modify the framework based on the objective and context.
- **Country ownership, government leadership and multi-partner involvement:** The Government of Bangladesh demonstrated leadership in forming and leading the national working group, chairing the workshops, developing the strategic plans, and bringing multiple partners together for building national consensus on introducing pulse oximetry in routine IMCI services in Bangladesh. All the major non-government organisations and development partners related to IMCI implementation in Bangladesh committed to using the updated National IMCI Implementation Package in their respective health system strengthening initiatives.
- **Expertise, experience and relationships of the facilitating organisation:** The technical expertise and experience of icddr,b in the introduction and scale-up of IMCI services in Bangladesh and pre-existing relationships with other high power-high interest stakeholder organisations substantially contributed to the identification, sensitisation, involvement, and engagement of other high power-high interest stakeholder organisations. Moreover, the communication, and organising skills of icddr,b contributed to facilitating the stakeholder engagement process efficiently. Therefore, the positionality of implementing organisations needs to be considered before adopting a particular approach for engaging stakeholders.

#### Weakness

- **Time-consuming:** It took more than two and a half years to implement the stakeholder engagement related activities and achieve the objective of incorporating the use of pulse oximetry in national policy and programme. Numerous workshops were organised based on the availability of all high power-high interest stakeholders, which was difficult considering their busy schedules, especially during ongoing COVID-19 pandemic. Extensive deskwork and preparations were necessary before organising these workshops, which called for several days or weeks of preparation. Hence, engaging the stakeholders effectively is time-consuming and will require patience and commitment by the facilitating organisation.
- **Resource-intensive:** The initial stage involved reviewing a significant number of documents to identify stakeholders and update the National IMCI Implementation Package, which required a substantial amount of person-time involvement of project staff. In addition, organising workshops at national, district and sub-district levels required numerous person-days of involvement from IMCI experts, managers, service providers and project staff. It also required extensive travelling by the project staff. Since stakeholder engagement is a resource-intensive process, it is important to have a planning and specific budgetary allocation for stakeholder engagement at the outset of any project.
- **Iterative:** Reviewing, editing, and formatting several lengthy documents were essential for updating the National IMCI Implementation Package. Some of these documents required numerous rounds of editing and formatting to address and incorporate the feedback of all the stakeholders across the national, district and sub-district levels before finalisation. For example, IMCI chart booklet had 23 rounds of iteration and IMCI registers had 19 rounds of iteration before finalisation. Hence, the facilitating organisation need to have dedication and attention to details to effectively steer the stakeholder engagement process.
- **Mercurial:** The power-interest matrix of the stakeholders does not stay static and could change at any given time. For example, during the engagement process, one of the stakeholders with high power and low interest unexpectedly turned into a high power-high interest actor and started posing a risk to the process. Therefore, some part of the stakeholder engagement process was adapted based on the agenda of that particular stakeholder. In addition, during the stakeholder engagement process, there can be changes in leadership positions. For example, the National Newborn Health and IMCI Programme manager was changed twice, and the district health manager was changed three times during the engagement process, owing to administrative transfers. It required investing additional time and efforts to sensitise the new stakeholders and garnering their active buy-in to introduce pulse oximetry in routine IMCI services. Hence, stakeholder engagement is an adaptive process and will require flexibility in revising, updating and changing the plan based on the context and circumstances.

#### Opportunity

- **COVID-19 pandemic- general awareness regarding hypoxaemia:** The COVID-19 pandemic has raised awareness among the health care provider and care receiver for using pulse oximetry in measuring the oxygen saturation level and assessing the severity of pneumonia. This opportunity has facilitated the implementation process more easily.

#### Threats

- **COVID-19 Pandemic- lockdown and restrictions:** The stakeholder engagement related activities and plans required extensive modification and revision due to the COVID-19 pandemic. During the first lockdown period between March and August 2020, all project-related activities were suspended. The team advocated with the National Newborn Health and IMCI Programme to resume project activities after the COVID-19 related restrictions were eased.
- **Other national and programme priorities:** The engagement process and project-related activities were also interrupted by unanticipated events, such as implementing the national Measles-Rubella campaign, which kept the district and sub-district health managers and IMCI service providers occupied during February and March 2021. Therefore, the potential impact of an emerging threat on the overall outcome and timeline of the stakeholder engagement process must be acknowledged and should have the flexibility of modifying the plan to take course corrective measures.

## CONCLUSIONS

Our experience has demonstrated the value and potential of the approach that we adopted for stakeholder engagement. Our engagement process contributed to the national decision to introduce pulse oximetry in routine IMCI services and update the national implementation package, which are steppingstones towards scalability and sustainability. However, our experience also reveals that stakeholder engagement is a context-driven, time-consuming, resource-intensive, iterative, mercurial process that demands planning, prioritisation, inclusiveness and adaptability. It is also influenced by the expertise, experience and positionality of the facilitating organisation. However, the approach needs to be conceptualised coupled with the allocation of adequate resources and time commitment to replicate its impact and implement it effectively in other settings.

## Additional material


Online Supplementary Document
